# Splenosis Mimicking Peritoneal Carcinomatosis

**DOI:** 10.5334/jbsr.2089

**Published:** 2020-04-06

**Authors:** Louise Pichon, Olivier Lebecque, Nicolas Mulquin

**Affiliations:** 1 Cliniques universitaires Saint-Luc, Bruxelles, BE; 2 Université catholique de Louvain, CHU UCL Namur, Department of Radiology, Yvoir, BE

**Keywords:** splenosis, computed tomography, splenectomy, scintigraphy

## Abstract

**Teaching point:** Splenosis must be considered in patients with history of splenectomy or splenic trauma, later presenting with multiple peritoneal nodules.

## Case Report

A 44-year-old man came to the emergency room for two-week progressive dyspnea. His medical history included morbid obesity, active smoking and childhood severe trauma that required splenectomy.

**Figure 1:**
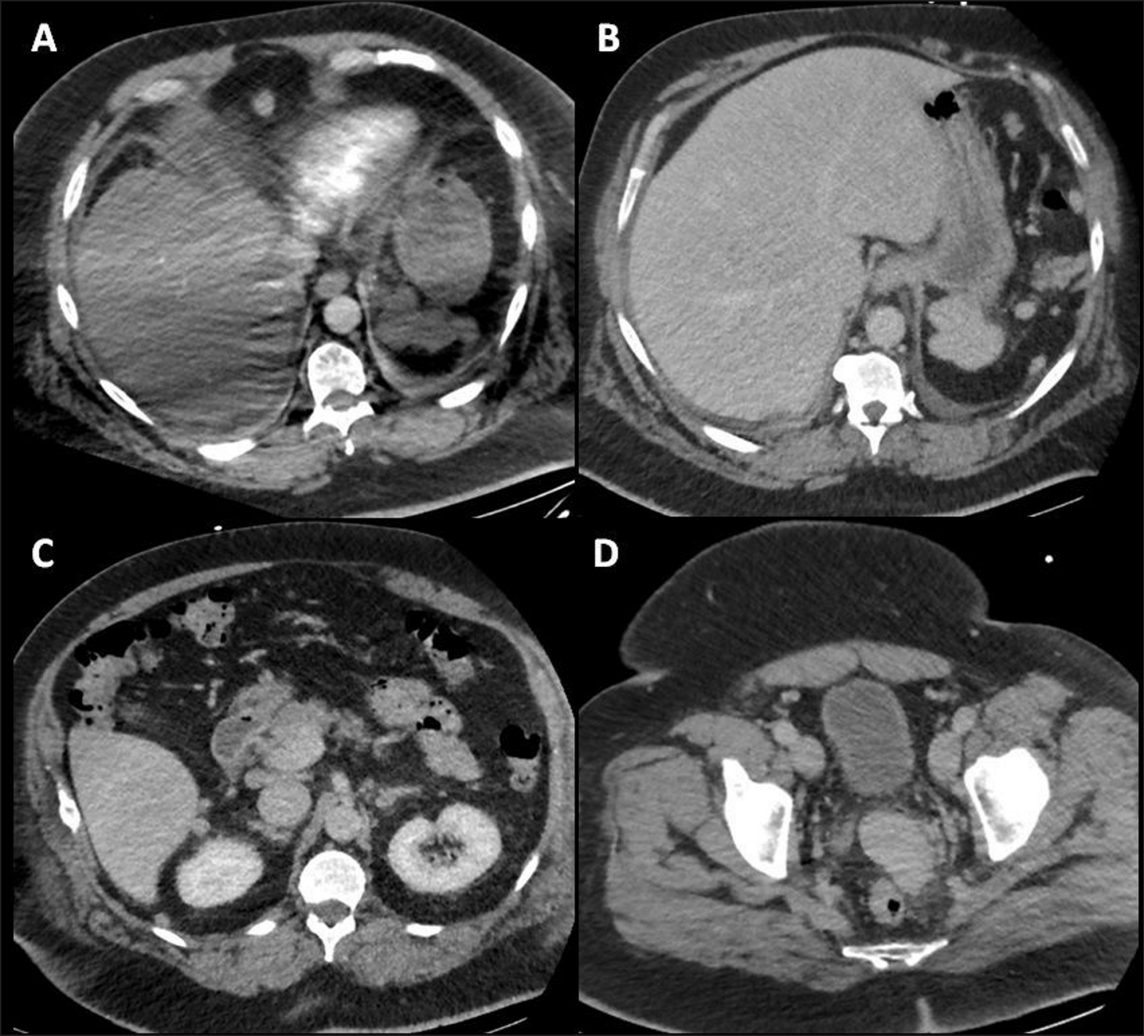
CT pulmonary angiography, arterial phase, axial slice **(A)** shows nodular lesions in the upper abdomen. Abdominal CT, portal venous phase, axial slice **(B–D)** shows multiples abdominopelvic nodular lesions.

The computed tomography (CT) pulmonary angiography performed to rule out a pulmonary embolism revealed several peritoneal nodules in the upper abdomen (Figure 1A).

**Figure 2:**
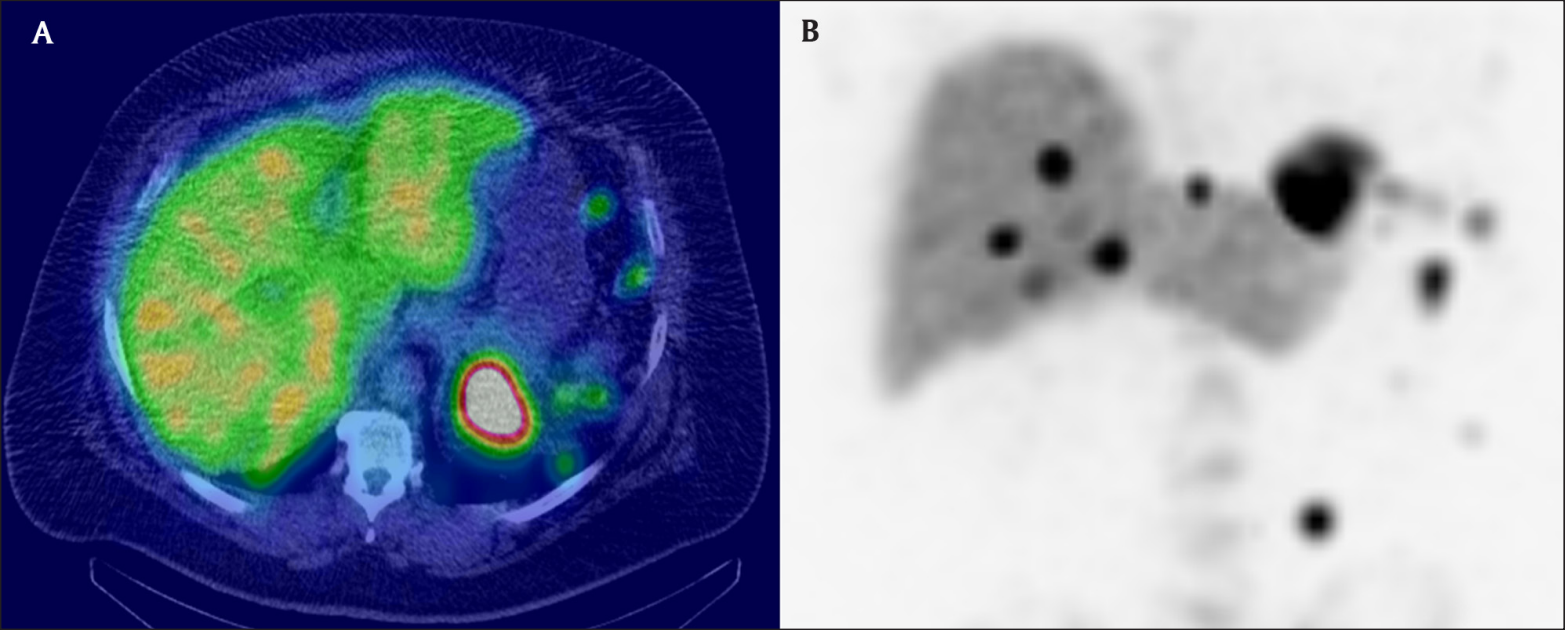
Fusion Tc99m-DRBC + CT axial slice **(A)** and coronal maximal intensity projection reconstruction of the scintigraphy **(B)** demonstrating uptake of these multiples nodular lesions.

The additional abdominal contrast-enhanced CT revealed multiple other nodules and homogeneous disseminated peritoneal masses (Figure 1B–C1). Splenosis nodules were suspected given the history of splenic trauma and the lack of cancer history. A Tc-99m-tagged heat-damaged red blood cells scintigraphy (Tc99m-DRBC) confirmed the diagnosis of peritoneal splenosis (Figure 2).

## Comment

Splenosis corresponds to an ectopic autotransplantation of splenic tissue. Unlike accessory spleens and polysplenia, this is an acquired anomaly; it occurs following a rupture of the splenic capsule (post-traumatic or iatrogenic) with direct seeding of splenic tissue or haematogenous propagation.

Splenosis nodules are mostly found in the abdominal cavity but distant localizations have also been described. As a benign entity, splenosis is most often asymptomatic and discovered incidentally. Therapeutic abstention is the rule, except in rare symptomatic cases.

On imaging, splenosis nodules appear well defined and homogeneous. Nevertheless, splenosis can mimic primary or metastatic tumor lesions (notably peritoneal carcinomatosis) or endometriosis.

On MRI, they have the same signal characteristics as normal spleen tissue; hypointense on T1-weighted images and hyperintense on T2-weighted images relative to the liver.

On CT, these nodules are isodense compared to a native spleen.

The ultrasound appearance is not very specific, the nodules are discretely hypoechoic compared to the liver and present a posterior acoustic enhancement.

Whatever the imaging technique, the enhancement kinetics of splenosis nodules is similar to that of healthy splenic parenchyma: intense enhancement, heterogeneous in the arterial phase (“Zebra spleen”), homogenizing in the portal phase.

Tc99m-DRBC is the best examination method to confirm the diagnosis of splenosis, as the ectopic splenic tissue absorbs most of the damaged red blood cells [1]. It is much more sensitive and more specific than Tc-99m-labeled sulfur colloid scintigraphy.

In the event of the discovery of isolated or multiple nodules in a patient with a history of trauma or splenic surgery, splenosis must be part of the differential diagnosis in order to avoid any unnecessary additional invasive procedure.
